# Identification of Volatile Components of Liverwort (*Porella cordaeana*) Extracts Using GC/MS-SPME and Their Antimicrobial Activity 

**DOI:** 10.3390/molecules17066982

**Published:** 2012-06-06

**Authors:** Danka Bukvicki, Davide Gottardi, Milan Veljic, Petar D. Marin, Lucia Vannini, Maria Elisabetta Guerzoni

**Affiliations:** 1 Institute of Botany and Botanical Garden “Jevremovac”, Faculty of Biology, University of Belgrade, Studentski trg 16, 11000 Belgrade, Serbia; Email: veljicm@bio.bg.ac.rs (M.V.); pdmarin@bio.bg.ac.rs (P.D.M.); 2 Department of Food Science, University of Bologna, Via Fanin 46, 40127 Bologna, Italy; Email: da.gottardi@gmail.com (D.G.); lucia.vannini2@unibo.it (L.V.); elisabetta.guerzoni@unibo.it (M.E.G.)

**Keywords:** liverwort, *Porella cordaeana*, GC/MS-SPME, antimicrobial activity, food microorganisms

## Abstract

Chemical constituents of liverwort (*Porella cordaeana*) extracts have been identified using solid-phase microextraction-gas chromatography mass spectrometry (SPME-GC/MS). The methanol, ethanol and ethyl acetate extracts were rich in terpenoids such as sesquiterpene hydrocarbons (53.12%, 51.68%, 23.16%), and monoterpene hydrocarbons (22.83%, 18.90%, 23.36%), respectively. The dominant compounds in the extracts were β-phellandrene (15.54%, 13.66%, 12.10%) and β-caryophyllene (10.72%, 8.29%, 7.79%, respectively). The antimicrobial activity of the extracts was evaluated against eleven food microorganisms using the microdilution and disc diffusion methods. The minimum inhibitory concentration (MIC) varied from 0.50 to 2.00 mg/mL for yeast strains (*Saccharomyces cerevisiae 635*, *Zygosacharomyces bailii 45*, *Aerobasidium pullulans L6F*, *Pichia membranaefaciens OC 71*, *Pichia membranaefaciens OC 70*, *Pichia anomala* *CBS 5759*, *Pichia anomala DBVPG 3003* and *Yarrowia lipolytica RO13*), and from 1.00 to 3.00 mg/mL for bacterial strains(*Salmonellaenteritidis 155*, *Escherichia coli 555* and *Listeria monocytogenes 56Ly*). Methanol extract showed better activity in comparison with ethanol and ethyl acetate extracts. High percentages of monoterpene and sesquiterpene hydrocarbons could be responsible for the better antimicrobial activity.

## 1. Introduction

Bryophytes possess some bioactive components and therefore are used throughout the World as drugs and remedies to cure various diseases [1]. They have been proven to be rich sources of antimicrobials, and attempts to find potent, nontoxic, broad-spectrum active molecules have been widely undertaken. Liverwort extracts with antimicrobial effects are even sold commercially [2]. They are used in pharmaceutical products, horticulture and good indicators of environmental conditions.

Phytochemical studies of liverworts have shown that they contain a wide variety of structurally interesting compounds, some of which are antibacterial, antifungal, anticancer and diuretic agents [3]. Liverworts contain a large amount of mono-, sesqui- and diterpenoids, and aromatic compounds. In particular, they contain pinguisane-type sesquiterpenoids, sacculatane-type diterpenoids and bis(bi-benzyl) aromatic compounds which have not been found in higher plants [4]. 

Previous studies on the chemical composition of liverworts revealed that they often produce unique sesquiterpenoids with novel carbon skeletons, which are not isolated from vascular plants, and most of the sesquiterpenoids from liverworts correspond to enantiomers of those from higher plants. On the other side, a large variety of sesquiterpene hydrocarbons are important intermediates in the biosynthesis of functionalized sesquiterpenes, which may be useful reference compounds [5].

The cosmopolitan liverwort genus *Porella*, consisting of some 50–60 species, is widely distributed in temperate areas. *Porella* L. is characterised by bilobed, incubously inserted leaves with a vestigial, often recurved keel, large underleaves that are similar to the lobules in shape, terminal gynoecia on short lateral branches, large, flattened perianths, and a capsule that is barely exerted from the perianth and dehisces into numerous irregular valves [6].

In earlier work, volatile components and pinguisane-type sesquiterpenoids from American samples of *Porella cordaeana* were reported [7]. Three new pinguisane-type sesquiterpenoids, *i.e.*, porellapinguisanolide, porellapinguisenone and spiropinguisanin have been isolated, together with the previously known pinguisanin, norpinguisone methyl ester, striatenone and squalene. In more recent articles, it has been shown that the *Porella *species are also a rich source of terpenoid natural products including a range of pinguisanes [8]. Most *Porella* species are rich sources of sesqui- and diterpenoids, many of which show interesting biological activities [9]. Fifteen *Porella* species have been divided into nine types, drimane-aromadendrane-pinguisane-type (type I), sacculatane-type (II), pinguisane-type (III), pinguisane-sacculatane-type (IV), africanane-type (V), santalane-africanane-cyclofarnesane-type (VI), guaiane-type (VII), germacrane-pinguisane-sacculatane-type (VIII) and germacrane-africanane-guaiane-type (IX) [3]. *P. cordaeana* (type IV), collected in Europe produces mainly striatanes and pinguisanes and sacculatanes [3].

To the best of our knowledge there are no reports on the GC/MS-SPME analysis and antimicrobial properties of the extracts from the liverwort *P. cordaeana. *The aim of this study was to analyze volatile compounds of different extracts of *P. cordaeana *by using the GC/MS-SPME technique and evaluate the extracts’ activity against important food spoilage and potential pathogenic microorganisms.

## 2. Results and Discussion

### 2.1. GC/MS-SPME Analysis of the Chemical Composition of the Extracts

Qualitative and quantitative analysis of extracts are listed in Table 1. Among the groups, sesquiterpene hydrocarbons predominated (53.12%, 51.68%, 23.16%), followed by monoterpene hydrocarbons (22.83%, 18.90%, 23.36%), non-terpene hydrocarbons (0.57%, 0.96%, 42.90%) and alcohols (5.32%, 18.34%, 3.54%) in methanol, ethanol and ethyl acetate extracts, respectively. 

**Table 1:** Chemical composition of *P. cordaeana* extracts.

Extract		^a^ MeOH	^b^ EtOH	^c^ EtOAc
Compounds	RI	%	%	%
**Monoterpene hydrocarbons**				
Camphene	1077	0.11	0.10	0.33
β-Myrcene	1167	0.00	0.00	1.46
α-Phellandrene	1177	0.84	0.40	1.37
D-Limonene	1208	3.68	2.93	3.31
β-Phellandrene	1220	15.54	13.66	12.10
*m* -Cymene	1267	2.66	1.81	1.68
*p* -Cymene	1280	0.00	0.00	3.11
Totals:		**22.83**	**18.90**	**23.36**
**Sesquiterpene hydrocarbons**				
α-Cubebene	1463	0.11	0.10	0.00
δ-Elemene	1472	0.38	0.22	0.00
β-Himachalene	1498	0.53	0.17	0.00
Cycloseychellene	1513	3.87	4.45	2.87
α-Farnesene	1524	0.42	0.47	0.00
β-Cubebene	1531	0.31	0.46	0.00
β-Humulene	1538	0.64	0.63	0.00
α-Longipinene	1552	0.64	0.88	0.00
α-Santalene	1557	0.98	1.14	0.18
β-Elemene	1571	3.94	2.77	1.39
ç-Gurjunene	1583	3.27	5.15	2.21
Neoisolongifolene	1588	5.60	6.25	4.14
β-Cedrene	1590	1.46	1.60	0.23
β-Caryophyllene	1594	10.72	8.29	7.79
*cis* -Thujopsene	1623	3.98	1.60	0.00
Aromadendrene	1652	3.43	2.81	0.14
β-Farnesene	1664	0.15	0.00	0.11
γ-Muurolene	1671	3.48	3.68	2.24
di-epi-α-Cedrene	1677	0.44	0.71	0.00
Sesquichamene	1692	0.44	0.54	0.00
Aciphyllene	1701	0.00	0.14	0.00
Germacrene	1717	1.10	0.00	0.22
Valencene	1726	3.51	4.06	0.45
Elixene	1746	0.09	0.16	0.00
α-Chamigrene	1755	2.36	2.89	0.24
α-Curcumene	1776	0.13	0.11	0.09
(−)-α-Panasinsene	1784	1.07	1.21	0.13
Cuparene	1851	0.00	1.10	0.08
Calamenene	1855	0.07	0.00	0.65
Spathulenol	2129	0.00	0.09	0.00
Totals:		**53.12**	**51.68**	**23.16**
**Non-terpene hydrocarbons**				
3-Methylnonane	965	0.00	0.00	0.22
Decane	984	0.00	0.00	2.80
3,7-Dimethylnonane	1008	0.00	0.00	0.67
5-Methyldecane	1037	0.00	0.00	1.28
2-Methyldecane	1038	0.00	0.87	3.69
3-Methyldecane	1048	0.00	0.00	3.17
Undecane	1091	0.00	0.09	5.32
2,3-Dimethyldecane	1142	0.00	0.00	0.91
Dodecane	1190	0.00	0.00	1.44
*m*-Ethyltoluene	1230	0.00	0.00	0.67
Mesitylene	1253	0.00	0.00	0.22
Hemimellitene	1292	0.00	0.00	3.40
*m*-Diethylbenzene	1308	0.00	0.00	0.31
*m* -Propyltoluene	1315	0.00	0.00	1.05
*p* -Propyltoluene	1318	0.00	0.00	0.40
Butylbenzene	1322	0.00	0.00	0.31
4-Ethyl-*m* -xylene	1337	0.00	0.00	1.46
1,2-Diethylbenzene	1342	0.00	0.00	0.71
*o* -Propyltoluene	1350	0.00	0.00	2.89
2-Ethyl-*p* -xylene	1368	0.00	0.00	3.13
2-Ethyl-1,3-dimethylbenzene	1375	0.57	0.00	4.32
Cumene	1384	0.00	0.00	1.04
3,5-Diethyltoluene	1402	0.00	0.00	1.30
Durene	1488	0.00	0.00	1.44
5-Methylindane	1510	0.00	0.00	0.45
1,6-Dimethylindane	1527	0.00	0.00	0.30
Totals:		**0.57**	**0.96**	**42.90**
**Esters**				
Acetic acid methyl ester	823	1.21	0.00	0.00
Pyruvic acid methyl ester	1240	0.05	0.00	0.00
Totals:		**1.26**	**0.00**	**0.00**
**Alcohols**				
1-Propanol	1037	0.00	0.46	0.00
1-Butanol	1142	0.00	0.09	0.00
1-Ethoxy-2-propanol	1168	0.00	1.46	0.00
2-Methyl-1-butanol	1202	0.27	8.24	0.00
4-Methyl-2-pentanol	1162	4.20	5.60	3.54
1-Propoxy-2-propanol	1245	0.20	1.54	0.00
1-Hexanol	1348	0.39	0.95	0.00
1-Octen-3-ol	1441	0.26	0.00	0.00
Totals:		**5.32**	**18.34**	**3.54**
**Ketones**				
4-Methyl-3-pentene-2-one	1146	0.57	0.30	0.00
Methyl isobutyl ketone	1007	0.75	0.00	0.00
Totals:		**1.32**	**0.30**	**0.00**
**Aldehydes**				
Pentanal	982	0.51	0.21	0.00
2-Butenal	1049	0.00	0.09	0.00
2-Methyl-1-propanal	1092	0.00	0.17	0.00
Hexanal	1097	0.99	0.52	0.00
Totals:		**1.50**	**0.99**	**0.00**
**Others**				
2-Pentylfuran	1235	0.05	0.00	0.00
Acetic acid	1448	8.02	4.77	5.57
Propanoic acid	1519	3.73	2.29	0.00
Hexanoic acid	1861	0.34	0.22	0.00
Totals:		**12.14**	**7.28**	**5.57**
Unidentified		1.94	1.55	1.47
Total identified %		98.06	98.45	98.53

RI = Retention Index on CP WAX 52 CB capillary column; ^a^ MeOH: methanol extract; ^b^ EtOH: ethanol extract; ^c^ EtOAc: ethyl acetate extract.

Esters, ketones and aldehydes were present in amounts lower than 3.00%. Forty-eight components were identified from the methanol extract of *P. cordaeana*, representing 98.06% of the total extract, and major compounds were β-phellandrene (15.54%), β-caryophyllene (10.72%) and acetic acid (8.02%). Forty-seven components were identified from the ethanol extract, representing 98.45% of the total extract, and β-phellandrene (13.66 %), β-caryophyllene (8.29%), 2-methyl-1-butanol (8.24%), neoisolongifolene (6.25%) and ç-Gurjunene (5.15%) were the major components. β-phellandrene (12.10%), β-caryophyllene (7.79%), undecane (5.32%), acetic acid (5.57%), D-limonene (3.31%), neoisolongifolene (4.14%) and *p*-Cymene (3.11%) were the main compounds in the ethyl acetate extract. 

**Figure 1:**
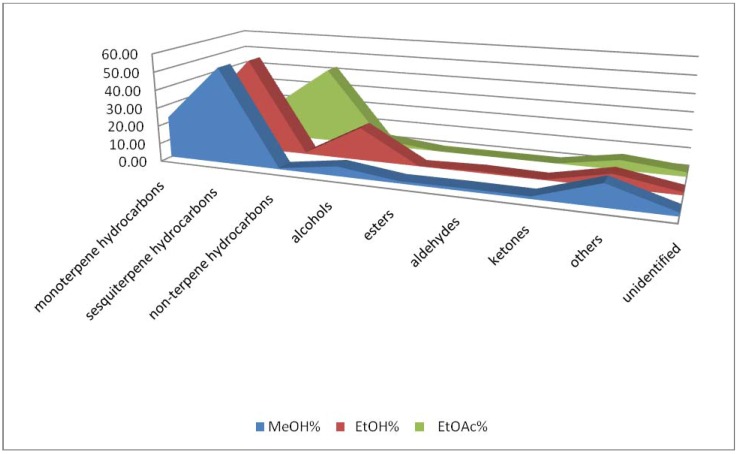
The chemical class distribution of the *P. cordaeana* extracts.

In the methanol extract 53.12% sesquiterpene hydrocarbons, 23.83% monoterpene hydrocarbons, 1.26% esters, 1.32% ketones and 5.32% alcohols were present. However, 1.94% of total compounds were not identified. In the *P. cordaeana* ethanol extract 51.68% sesquiterpene hydrocarbons, 18.90% monoterpene hydrocarbons, 0.30% ketones and 18.34% alcohols were identified, while in the ethyl acetate 23.16% sesquiterpene hydrocarbons, 23.36% monoterpene hydrocarbons, 42.90% non-terpene hydrocarbons and 3.54% alcohols were detected (Figure 1).

Several researchers have also reported that some *Porella* species contained sesquiterpenes [9-15].

### 2.2. Antimicrobial Activity

According to the results given in Table 2, all three extracts of *P. cordaeana* showed inhibition effects on the growth of all the strains tested. In particular, a notable antimicrobial activity was observed against all the strains of the tested yeasts species, which were more sensitive than bacteria to the effect of the extracts. For the yeast strains the MIC varied from 0.50 mg/mL to 2.00 mg/mL, while MFC varied from 1.00 mg/mL to 3.00 mg/mL. The most sensitive yeast was *Yarrowia lipolytica RO13* with a MIC of 0.50–1.00 mg/mL and a MFC of 1.5 mg/mL.* Zygossacharomyces bailii 45 *(MIC of 1.50–2.00 mg/mL and MFC of 3.00 mg/mL) and *P. membranaefaciens OC 70 *(MIC 2.00 mg/mL and MFC 2.50 mg/mL) were the most resistant ones to the extracts tested. The strongest anti-yeast activity was displayed by the methanol extractof *P. cordaeana. *Commercial antibiotic cycloheximideshowed inhibition for concentrations lower than 0.05 mg/mL for all micromycetes except against *Y. lipolytica RO13 *that was completely inhibited at 0.02 mg/mL (Table 2). The methanol extract exhibited the highest activity against *Y. lipolytica RO13.* In general the methanol extract was the most effective against all the strains (bacteria and yeasts). Methanol extract contained high amount (8.02%) of acetic acid which has shown excellent bactericidal effect [16].

**Table 2:** Antimicrobial activity of *P. cordaeana* methanol, ethanol and ethyl acetate extracts (mg/mL).

Liverwort Extracts	*P. cordaeana* MeOH extract		*P. cordaeana* EtOH extract		*P. cordaeana* EtOAc extract		Cycloheximide
Yeast	MIC	MFC	MIC	MFC	MIC	MFC	MIC
***S. cerevisiae 635***	1.25	2.00	1.50	2.00	1.50	2.00	<0.05
***Z. bailii 45***	2.00	3.00	1.50	3.00	1.50	3.00	<0.05
***A. pullulans L6F***	1.00	1.50	1.00	2.00	1.00	2.00	<0.05
***P.membranaefaciens OC 71***	1.25	2.00	2.00	2.50	2.00	2.50	<0.05
***P. memb. OC 70***	2.00	2.50	2.00	2.50	2.00	2.50	<0.05
***P. anomala*** *** CBS 5759***	1.00	1.50	1.00	1.50	1.00	1.50	<0.05
***P. anomala*** *** DBVPG 3003***	1.75	2.00	1.75	2.00	1.25	2.00	<0.05
***Yarrowia lipolytica RO13***	0.50	1.50	0.50	1.00	1.00	1.50	0.02

MIC: Minimal inhibitory concentration; MFC: Minimal microbicidal concentration.

Data relative to the antibacterial effects of *P. cordaeana* are presented in Table 3. The data showed the higher antimicrobial efficacy of methanol extract than the other two. MIC ranged between 1.00–3.00 mg/mL, while MBC was 1.50–3.00 mg/mL. The most sensitive bacterium was *Listeria monocytogenes 56Ly*. In particular, the methanol extract of *P. cordaeana *presented a MIC of 1.00 mg/mLagainst *L. monocytogenes 56Ly*.Furthermore, the growth of *L. monocytogenes 56Ly* was completely inhibited at 1.50 mg/mL for the methanol extract. The most resistant bacterial strain was *E. coli* 555 with a MIC of 2.00–3.00 mg/mL and MBC of 3.00 mg/mL. MIC for streptomycin was 0.02–0.05 mg/mL and MBC 0.1 mg/mL. According to the MICs and MBCs, the Gram-negative bacterial strains were more resistant than the Gram-positive one. *S. enteritidis 155 *and *E. coli 555 *(Gram-negative) showed MICs of 1 mg/mL, 2.00 mg/mL and MBCs of 2.00 mg/mL, 3.00 mg/mL respectively, for the methanol extract. Nevertheless, it should be taken into consideration that quite a high variability could be found in the sensitivity to antimicrobials among strains of the same microbial species. 

**Table 3:** Antibacterial activity of *P. cordaeana *methanol, ethanol and ethyl acetate extracts (mg/mL).

Liverwort Extracts	*P. cordeana* MeOH extract		*P. cordeana* EtOH extract		*P. cordeana* EtOAc extract		Streptomycin	
Bacteria	MIC	MBC	MIC	MBC	MIC	MBC	MIC	MBC
***S*. Enteritidis *155***	1.00	2.00	1.50	2.00	1.50	2.00	0.05	0.10
***L. monocytogenes 56Ly ***	1.00	1.50	1.50	2.00	1.50	2.00	0.02	0.10
***E. coli 555***	2.00	3.00	3.00	3.00	2.00	3.00	0.05	0.10

MIC: Minimal Inhibitory concentration; MBC: Minimal Bactericidal concentration.

**Figure 2:**
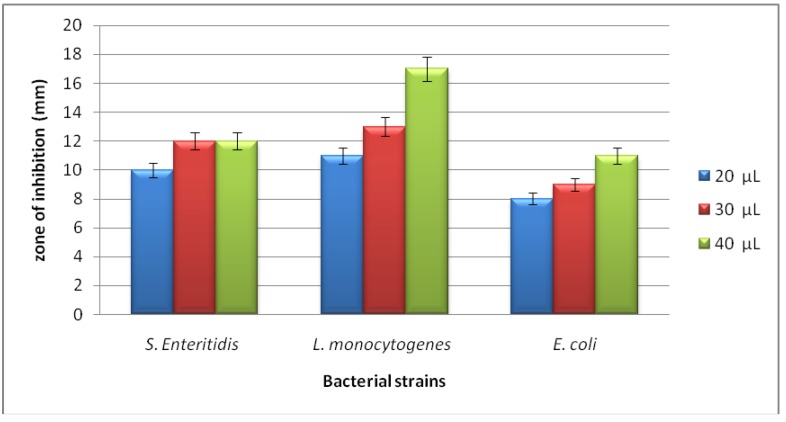
Zone of inhibition (mm) due to different concentrations (20, 30 and 40 µL) of *P. cordaeana* methanol extract against the bacterial strains of *S.* Enteritidis *155*, *E. coli 555* and *L. monocytogenes 56Ly*. Column height represents the mean of triplicate results and error bar represents the range of the results.

**Figure 3:**
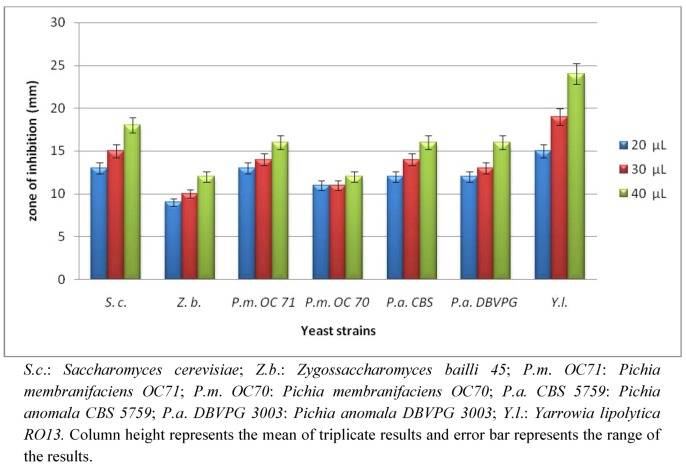
Zone of inhibition (mm) due to different concentrations (20, 30 and 40 µL) of *P. cordaeana* methanol extract against different yeast strains.

Results obtained from disc diffusion method followed by measurements of minimal inhibitory concentration (MIC), confirmed that *Y. lipolytica RO13* was the most sensitive microorganism with the highest inhibition zone (24 mm) (Figure 3) and the lowest MIC value (0.5 mg/mL) (Table 2). The results demonstrated that *Porella* methanol extract was more effective against the yeast strains than the bacterial ones. The methanol extract of *Porella* showed the maximum zones of inhibition against yeast *Y. lipolytica RO13* (15, 18 and 24 mm), followed by *S*. *cerevisiae 635* (13, 16 and 18 mm) and *P. membaranifaciensOC71* (13, 14 and 16 mm), whereas *P. anomalaCBS 5759* and *P. anomalaBVPG 3003* showed the same results (12, 14 and 16 mm). On the other hand, *P. membranifaciensOC70* (11, 11 and 12 mm) and *Z. bailli 45* (9, 10 and 13 mm) presented the lowest inhibition haloes due to 20, 30 and 40 μL of *Porella* extract. *Z. bailli* was the most resistant yeast (zone of inhibition was 9 mm), whereas *Y. lipolytica RO13* was the most susceptible also at the lowest concentration tested. 

The zones of inhibition detected for bacteria due to 40 μL of the methanol extract varied from 11 mm (*E. coli 555*) to 17 mm (*L. monocytogenes 56Ly*). Results summarized in Figure 2 indicate that *L. monocytogenes 56Ly* was the most susceptible bacterium for the three volumes of the extract (20, 40 and 60 μL). Based on the results of the chemical composition of the methanol extract it can be concluded that the antimicrobial activity is apparently attributed to its high content of sesquiterpenes and monoterpenes. *P. cordaeana* extracts are rich in sesquiterpenes hydrocarbons components as caryophyllene, neoisolongifolene, aromadendrene, γ-muurolene, and monoterpene hydrocarbons such as β-phellandrene, D-limonene and cymene. The results confirmed that the antimicrobial activity of *P. cordaeana *could be correlated to the presence of β-phellandrene and caryophyllene. These constituents have been reported to possess significant antimicrobial activity. Caryophyllene showed activity against the microorganisms *Escherichia coli*,* Staphylococcus aureus*,* Klebsiella pneumoniae*, *Pseudomonas aeruginosa *and *Candida albicans* [17]. 

In all the tested extracts β-phellandrene and caryophyllene were found as the major compounds which could be mainly responsible for the antimicrobial effect of the tested extracts. An antimicrobial activity of caryophyllene has been previously reported [18,19]. Plant extracts and essential oils that contain sesquiterpene hydrocarbons are reported to have high antimicrobial activity, and the monoterpenes found in the extract analysed in this work may act as antimicrobial agents [20].

Our results showed that *Porella* extracts could be used as natural preservative in food or pharmaceutical industries. Several liverworts display antifungal activity [21]. Moreover *Porella* extracts contain β-elemene (3.94%; 2.77%; 1.39% in methanol, ethanol and ethyl acetate extract, respectively), *i.e.*, compounds which in last recent years are known as strongly novel antitumor agent against ovarium cancer [22] and lung cancer [23]. D-limonene, which is a natural monocyclic monoterpene that has also been detected in significant percentages in all the *Porella* extracts (3.68%, 2.93%, 3.31%), has been shown to possess chemopreventive and therapeutic properties against many human cancers [24].

It is important to outline that the antibiotic cycloheximide, used in comparison with the *P. cordaeana *extracts and endowed with a strong activity against the test strains, is one of the most powerful antimicrobial agents. However, although it is widely used in biomedical research it is not suitable for human use both as therapeutic or antimicrobial agent as its health risks have become better known [25]. The bottleneck of the toxicity against humans and animals of the major part of the antimicrobial molecules or antibiotics delays antifungal discovery. The occurrence of antimicrobial compounds, which are known as safe for human consumption, in *P. cordaeana* could be successfully exploited particularly for the control of food borne yeasts or pathogenic yeasts like *Candida albicans.*

## 3. Experimental

### 3.1. Plant Material

The samples of *Porella cordaeana* (Hueb.) Moore were collected in April 2009, locality Zlatar Mt. (Serbia). A voucher specimen (No. 16620) has been deposited in the Herbarium at the Institute of Botany and Botanical Garden “Jevremovac”, University of Belgrade (BEOU). Material was dried at room temperature.

### 3.2. Extracts Preparation

Dried plants were pulverized into a fine powder using an electric blender. Powdered material (10 g) was extracted with methanol, ethanol or ethyl acetate (100 mL) for 24 h at room temperature. After 24 h, the mixture was filtered through Whatman No. 1 filter paper. Extracts were prepared on a rotary evaporator (Laborota 4001, Heidolph). The solvent was removed with a rotary vacuum evaporator at 40 °C. The yields of the extracts were 1.23%, 0.92% and 1.06% for methanol, ethanol and ethyl acetate extract, respectively. The obtained extracts were stored at 4 °C until further tests.

### 3.3. Gaschromatographic (GC)-Mass Spectrometry (MS)-Solid Phase Microextraction (SPME) Analysis

A divinylbenzene-poly(dimethylsiloxane)-coated stable flex fiber (65 μm) and a manual SPME holder (Supelco Inc., Bellefonte, PA, USA) were used in this study after preconditioning according to the manufacturer's instruction manual. Before each headspace sampling, the fiber was exposed to the GC inlet for 5 min for thermal desorption at 250 °C in a blank run.

Samples were put into sealed vials and then equilibrated for 10 min at 40 °C. The SPME fiber was exposed to each sample for 10 min by manually penetrating the septum, and, finally, the fiber was inserted into the injection port of the GC for 10 min sample desorption. GC-MS analyses were carried out on an Agilent 6890 gas chromatograph (Agilent Technologies, Palo Alto, CA, USA) coupled to an Agilent 5970 mass selective detector operating in electron impact mode (ionization voltage, 70 eV). A Chrompack CP Wax 52 CB capillary column (50 m length, 0.32 mm i.d., 1.2 μm df) was used (Chrompack, Middelburg, The Netherlands). The temperature program was 50 °C for 0 min, then programmed at 5 °C/min to 230 °C for 10 min. Injector, interface, and ion source temperatures were 250, 250, and 230 °C, respectively.

Injections were performed with a split ratio of 1:50 and helium (1 mL min^−1^) as the carrier gas. Identities were confirmed by searching mass spectra in the available databases (NIST, version 2005; Wiley, version 1996) and according to the Registry of Mass Spectral Data (1998) mass spectra libraries as well as literature MS data and in comparison with authentic chemical compounds when commercially available.

### 3.4. Chemicals and Strains

Different bacterial (*Salmonella* Enteritidis *155*, *Escherichia coli 555*–Gram-negative and *Listeria monocytogenes 56Ly*–Gram-positive) and yeast strains (*Saccharomyces cerevisiae 635*, *Zygosacharomyces bailii 45*, *Aerobasidium pullulans L6F*, *Pichia membranaefaciens OC 71*, *Pichia membranaefaciens OC 70* and *Yarrowia lipolytica RO13* belonging to the Department of Food Science, University of Bologna, Italy, as well as *Pichia anomala* CBS5759 and *Pichia anomala* *DBVPG 3003* were used to evaluate the effect of plant extracts. The yeasts and bacteria strains used in this study were grown on Yeast Extract Peptone Dextrose (YPD) and Tryptic Soy Broth (TSB) medium, respectively, at 27 °C for 48 h (yeasts) and 37 °C for 24 h (bacteria). Cells were suspended in sterile distilled water and used immediately.

### 3.5. Antimicrobial Assay

#### 3.5.1. Determination of Minimum Inhibitory Concentration (MIC) by Microdilution Method

In order to investigate the antimicrobial activity of the extracts, the modified microdilution technique was used [26,27]. Bacterial species were cultured overnight at 37 °C in a Tryptic Soy Broth (TSB) medium. Yeast strains were cultured at 27 °C in YPD medium. 

Minimal inhibitory concentrations (MICs) determination was performed by a serial dilution technique using 96-well microtitre plates. The investigated extracts were added in a broth TSB medium (bacteria)/broth YPD medium (yeasts) with inoculum. The microplates were incubated for 48 h at 37 °C for bacteria and 72 h at 28 °C for yeasts, respectively. The lowest concentrations without visible growth were defined as MIC the MBC (minimal bactericidal concentration)/MFC (minimal microbicidal concentration), indicating (99.5%) killing of the original inoculums, have been detected. Generally MBC/MFC values are defined as the minimal concentrations of the tested molecule not allowing the microbial growth when 10 µL of the cultures taken from the wells with no visible growth are plated onto agarised medium and incubated at the optimial temperatre. Dimethyl sulfoxide (DMSO) was used as an effective extraction solvent, and always used as negative control. All tests were performed in triplicate.

#### 3.5.2. Disc Diffusion Method

The following method was used: A portion of each suspension (100 μL) containing approximately 10^5^ cfu/mL was spread over the surface of TSB/YPD plates and allowed to dry. A paper disc (diameter 6 mm) was laid on the inside surface of the upper lid and 10 μL was placed on each disc. The plates inoculated with microorganisms were immediately inverted on top of the lid and sealed with parafilm to prevent leakage of the vapour. Plates were incubated at 37 °C/27 °C for 24 h/48 h and the diameter of the resulting inhibition zone in the bacterial/yeasts lawn was measured. The volume of extracts placed on the paper discs varied, *i.e.*, 20, 30 or 40 μL. A negative control was also included in the test using a filter paper disc saturated with DMSO to check possible activity of this solvent against the bacteria/yeasts assayed. All tests were performed in triplicate.

## 4. Conclusions

The results of this work demonstrated that *P. cordaeana* extracts possess significant antimicrobial potential against food microorganisms. The methanol extract showed a higher activity compared to ethanol and ethyl acetate ones. High percentages of monoterpene and sesquiterpene hydrocarbons could be responsible for antimicrobial activity. GC/MS-SPME analysis of volatile compounds showed some interesting substances which may prove useful in further investigations for food preservation purposes or as anticancer components.
